# Supplementary figures and images for: Viral RNA pUGylation promotes antiviral immunity in C. elegans
Source: J Virol. 2025 Oct 30;99(11):e01169-25. doi: 10.1128/jvi.01169-25 (PMC12645942; doi:10.1128/jvi.01169-25)

control

muscle

pan-soma

intestine

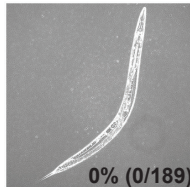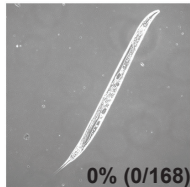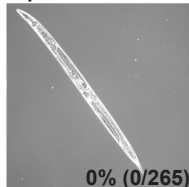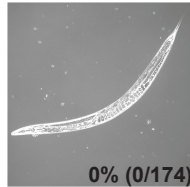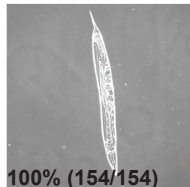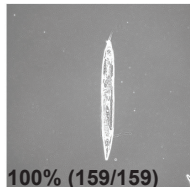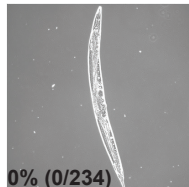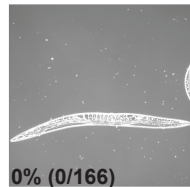

L4440 Control  
+ auxin

*dpy-6* RNAi  
+ auxin

# A

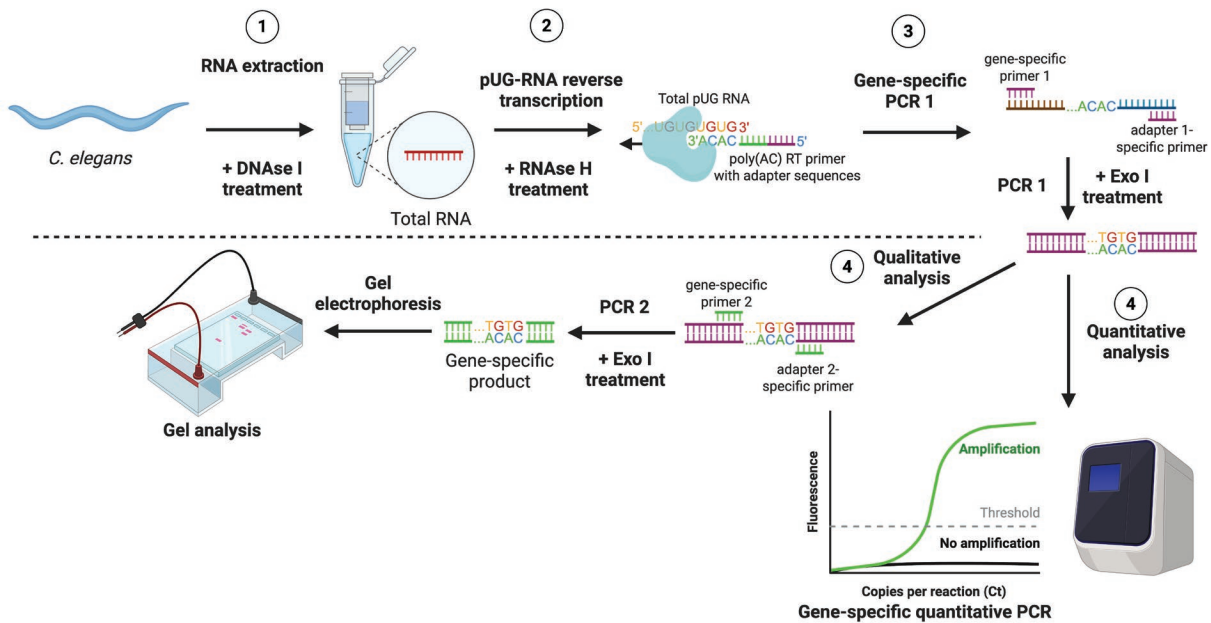

# B

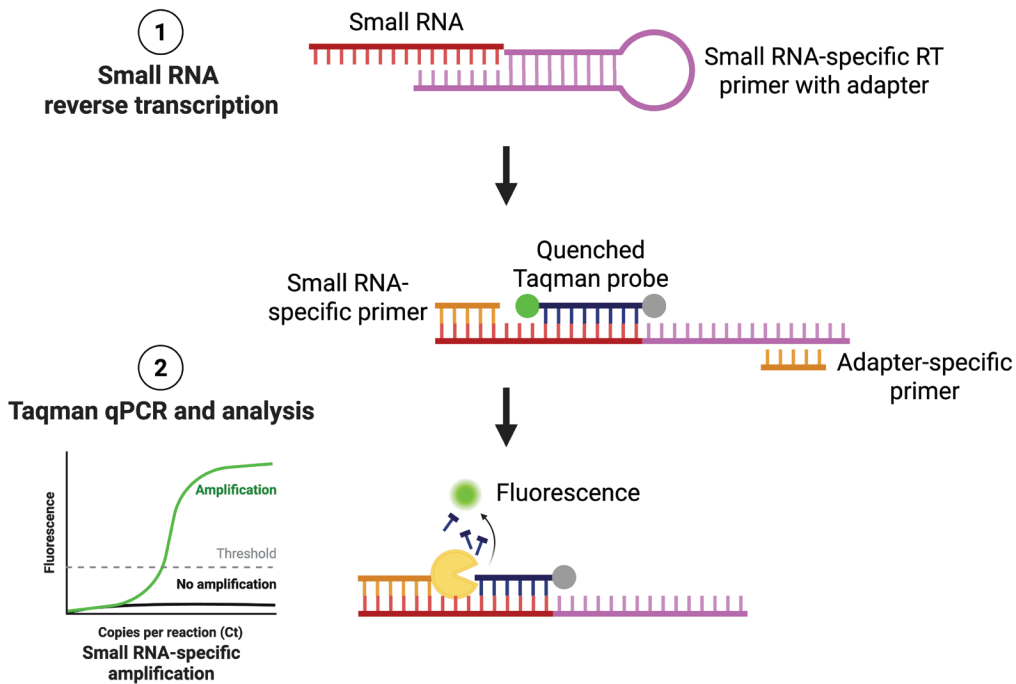

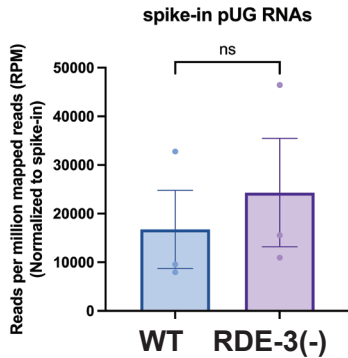

Supplement: Supplemental figures, part I — Figures S1 to S3. [file jvi.01169-25-s0001.pdf]

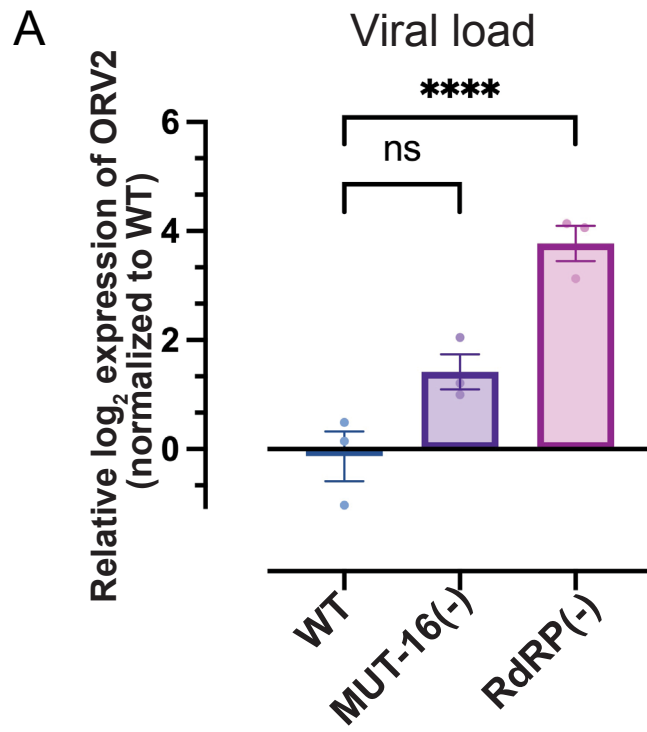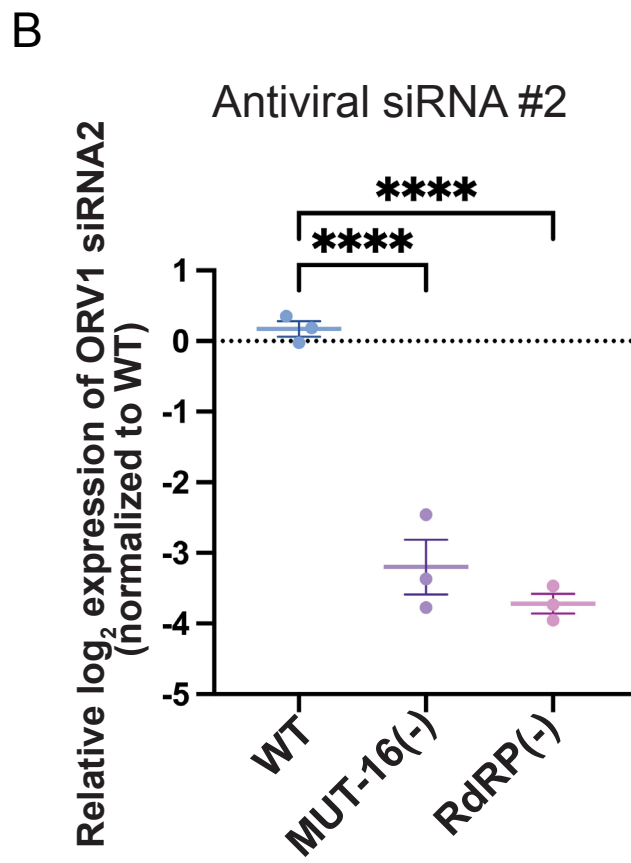

A

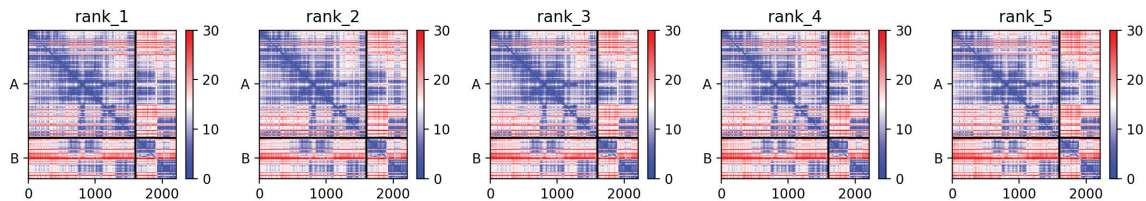

B

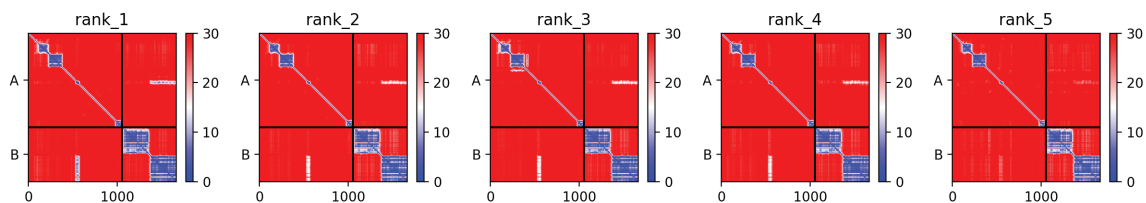

C

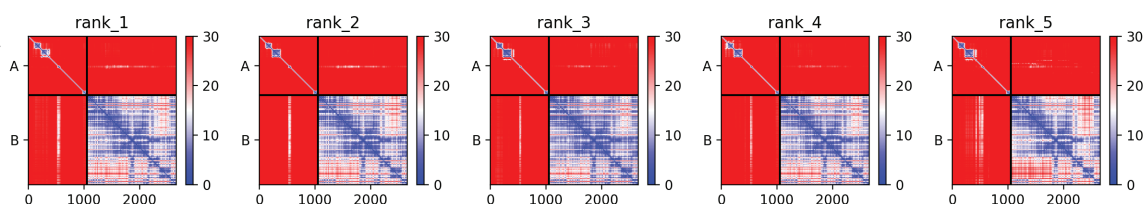

D

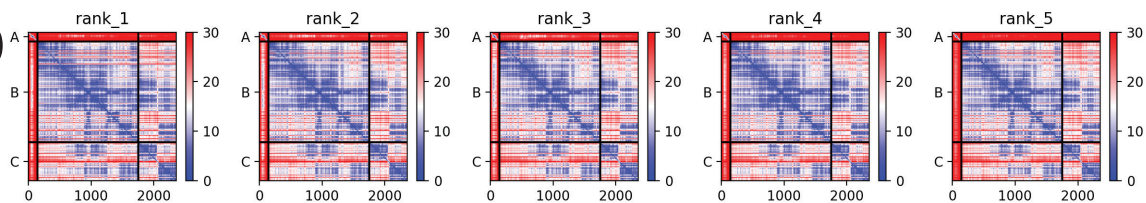

E

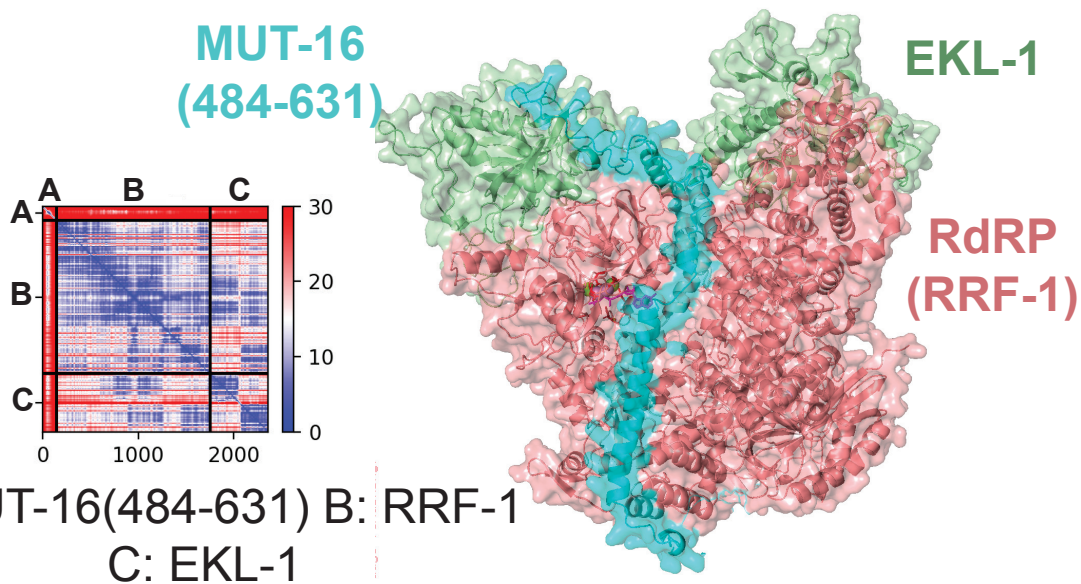

A

**pUGasome + MUT-16(strd)**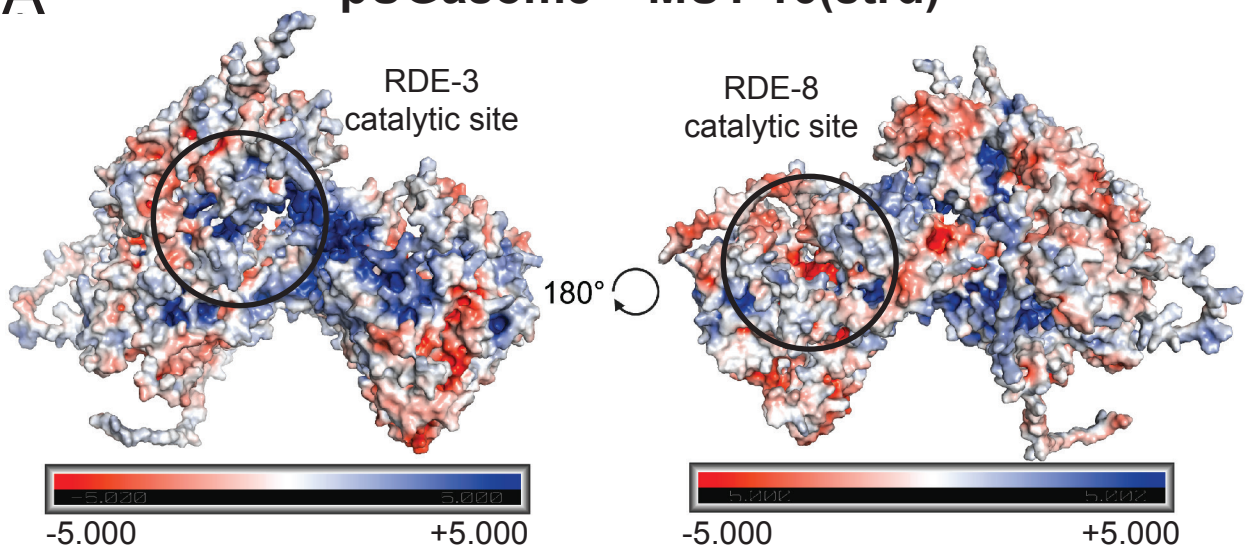

B

**pUGasome**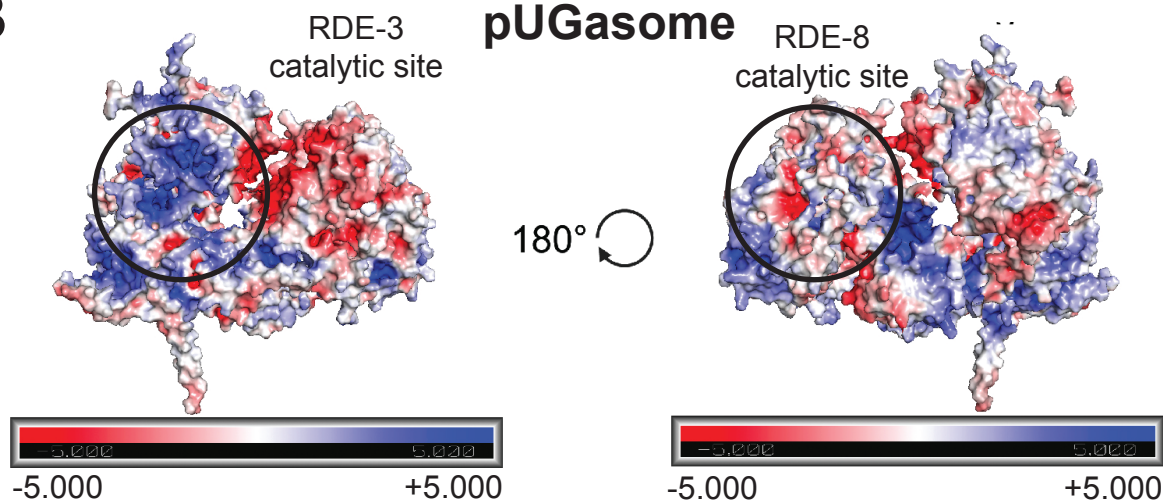

C

**pUGasome + pUG RNA**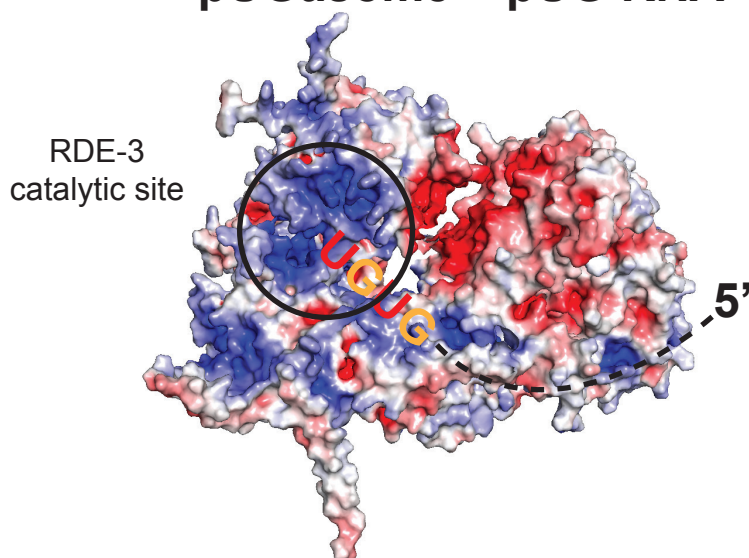

Supplement: Supplemental figures, part IV — Figures S11 to S13. [file jvi.01169-25-s0004.pdf]
