# Supplementary material for: Viral RNA pUGylation promotes antiviral immunity in C. elegans
Source: J Virol. 2025 Oct 30;99(11):e01169-25. doi: 10.1128/jvi.01169-25 (PMC12645942; doi:10.1128/jvi.01169-25)
Supplement: Supplemental figures, part II — Figures S4 to S6. [file jvi.01169-25-s0002.pdf]

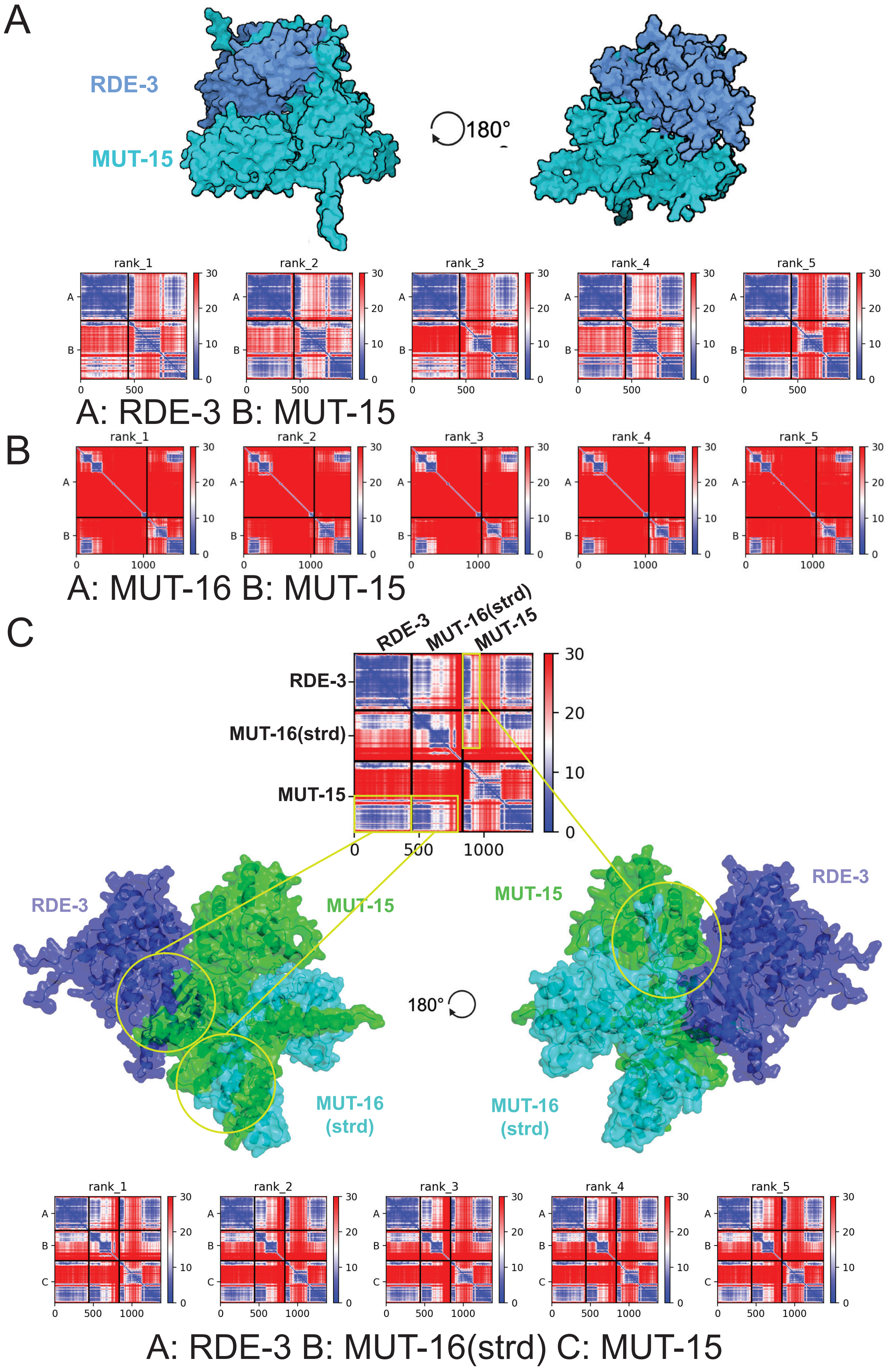

A
RDE-3
MUT-15
180°
rank_1
rank_2
rank_3
rank_4
rank_5
A: RDE-3 B: MUT-15
B
A: MUT-16 B: MUT-15
C
RDE-3
MUT-16(strd)
MUT-15
RDE-3
MUT-15
MUT-16 (strd)
180°
A: RDE-3 B: MUT-16(strd) C: MUT-15

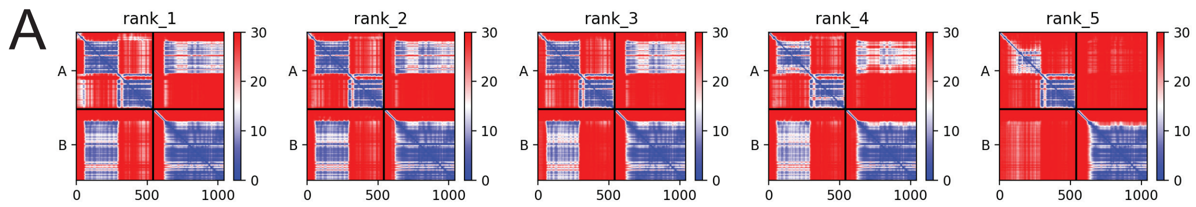

A: MUT-15 B: NYN-1

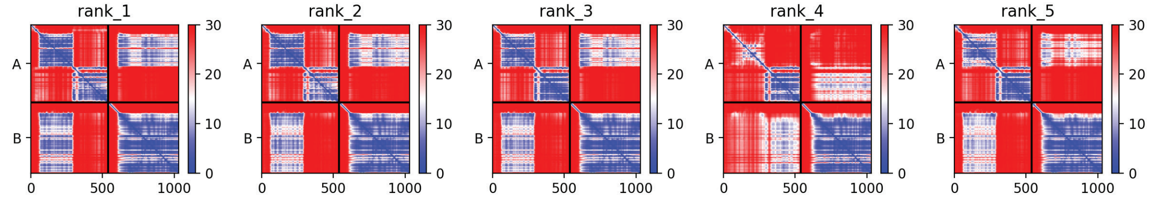

A: MUT-15 B: NYN-2

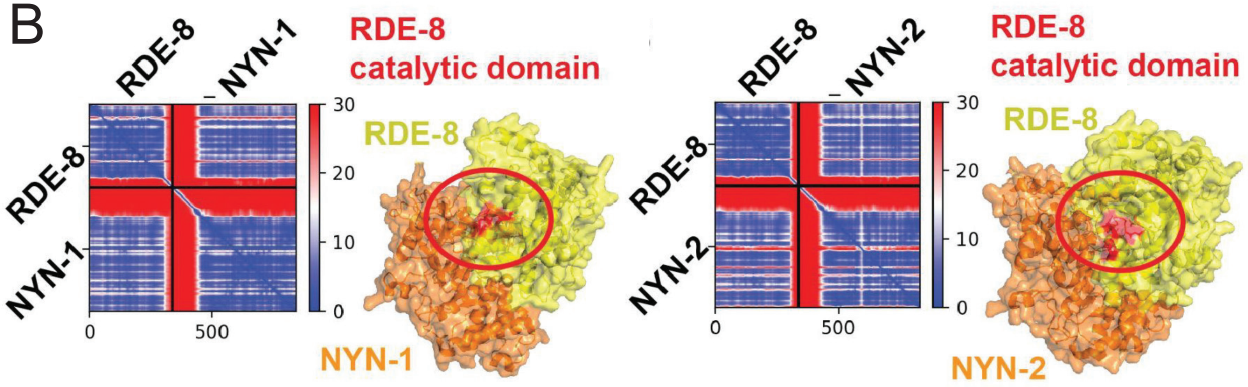

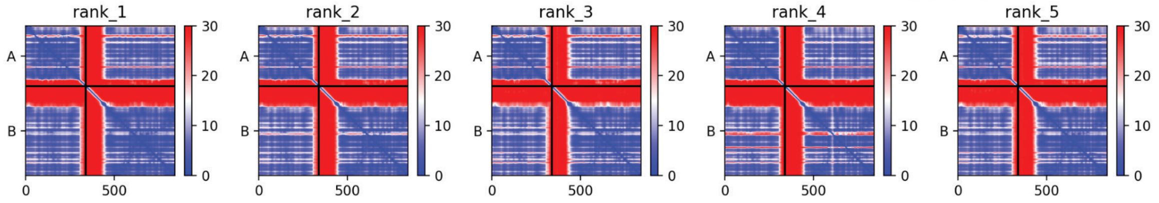

A: RDE-8 B: NYN-1

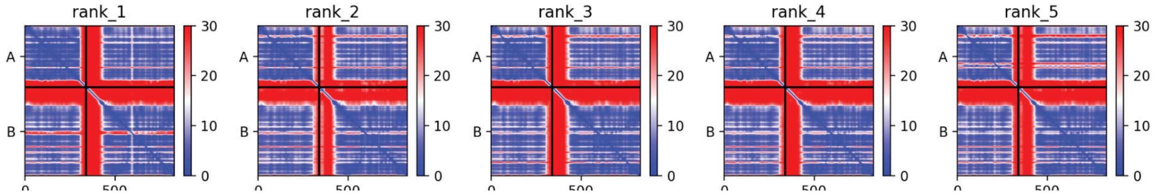

A: RDE-8 B: NYN-2

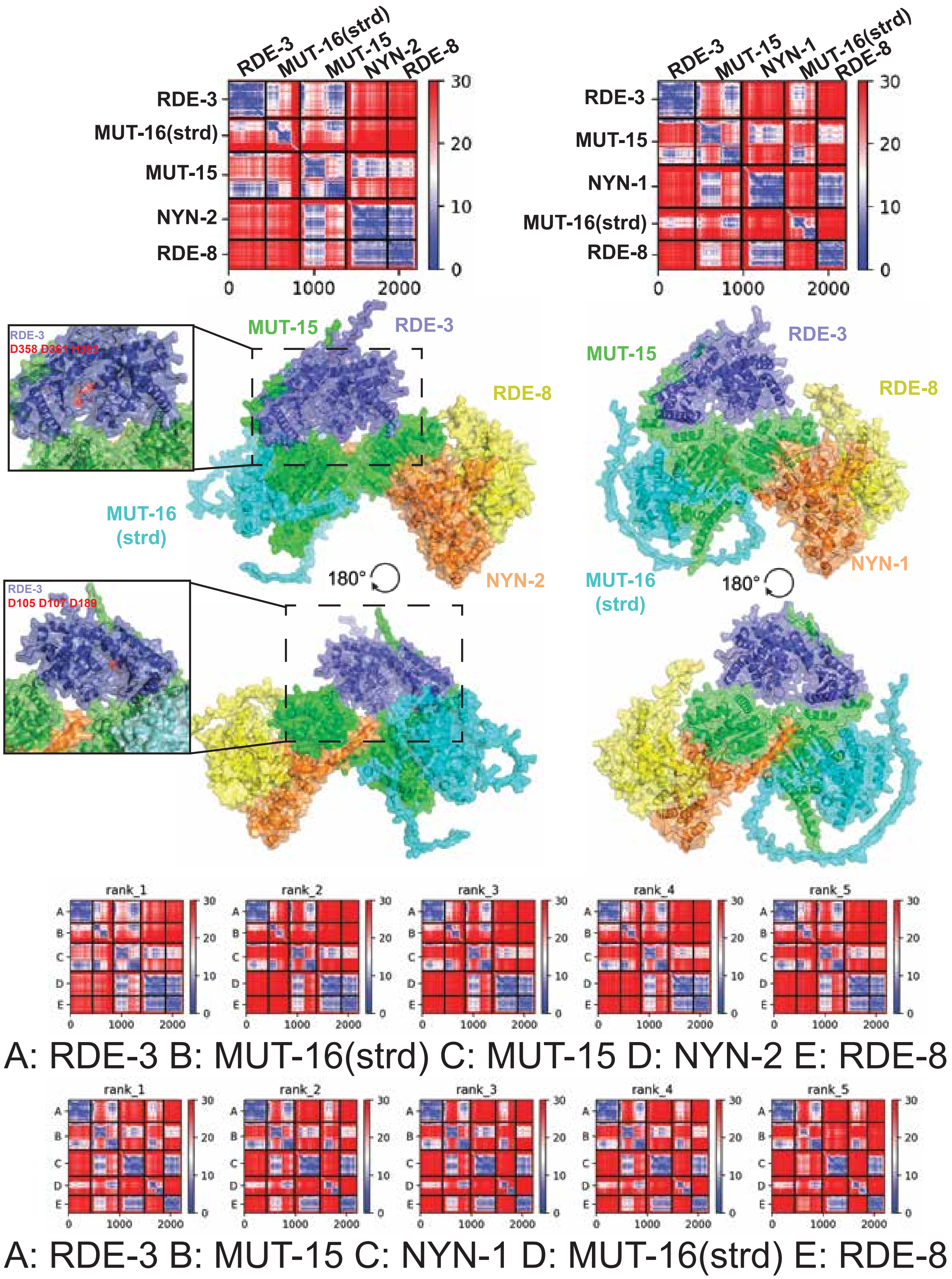

RDE-3
MUT-16(strd)
MUT-15
NYN-2
RDE-8
NYN-1
0
1000
2000
30
20
10
0
RDE-3
D358 D361 H363
D105 D107 D189
MUT-16
(strd)
180°
rank_1
rank_2
rank_3
rank_4
rank_5
A
B
C
D
E
A: RDE-3 B: MUT-16(strd) C: MUT-15 D: NYN-2 E: RDE-8
A: RDE-3 B: MUT-15 C: NYN-1 D: MUT-16(strd) E: RDE-8
