# Supplementary material for: Viral RNA pUGylation promotes antiviral immunity in C. elegans
Source: J Virol. 2025 Oct 30;99(11):e01169-25. doi: 10.1128/jvi.01169-25 (PMC12645942; doi:10.1128/jvi.01169-25)
Supplement: Supplemental figures, part III — Figures S7 to S10. [file jvi.01169-25-s0003.pdf]

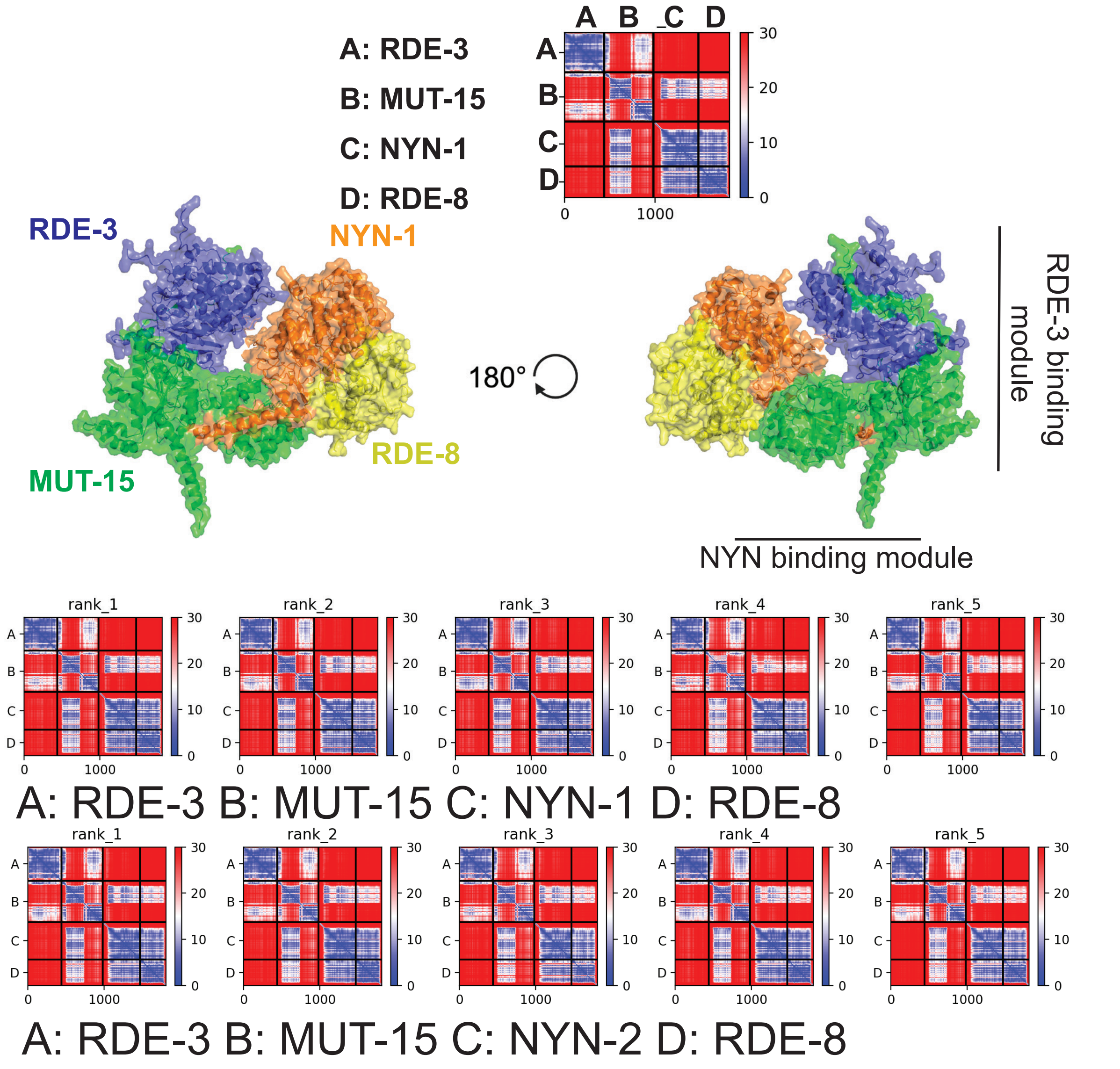

A: RDE-3
B: MUT-15
C: NYN-1
D: RDE-8
A B C D
30
20
10
0
0 1000
RDE-3
NYN-1
RDE-8
MUT-15
180°
RDE-3 binding module
NYN binding module
rank_1
rank_2
rank_3
rank_4
rank_5
A: RDE-3 B: MUT-15 C: NYN-1 D: RDE-8
rank_1
rank_2
rank_3
rank_4
rank_5
A: RDE-3 B: MUT-15 C: NYN-2 D: RDE-8

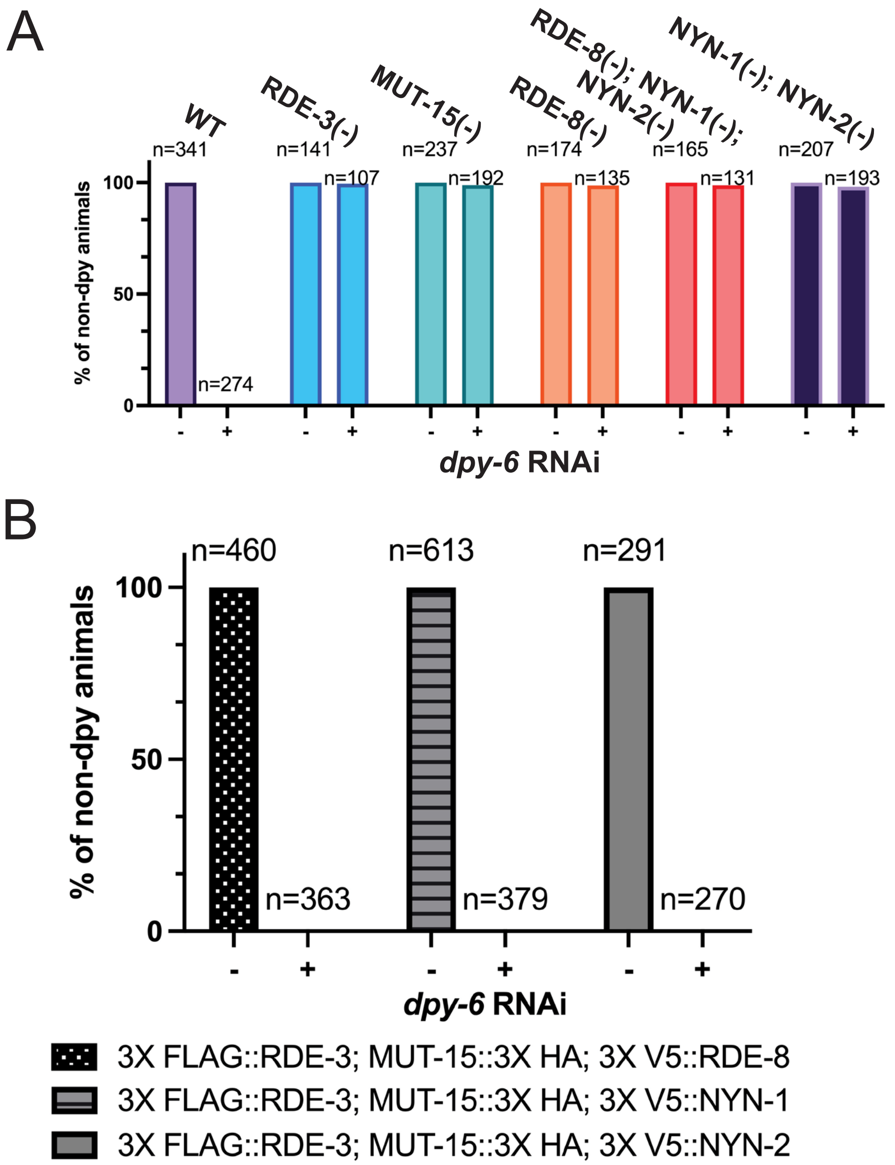
A
WT
RDE-3(-)
MUT-15(-)
RDE-8(-)
RDE-8(-); NYN-1(-); NYN-2(-)
NYN-1(-); NYN-2(-)
n=341
n=274
n=141
n=107
n=237
n=192
n=174
n=135
n=165
n=131
n=207
n=193
% of non-dpy animals
100
50
0
- + - + - + - + - + - +
dpy-6 RNAi
B
n=460
n=363
n=613
n=379
n=291
n=270
% of non-dpy animals
100
50
0
- + - + - +
dpy-6 RNAi
3X FLAG::RDE-3; MUT-15::3X HA; 3X V5::RDE-8
3X FLAG::RDE-3; MUT-15::3X HA; 3X V5::NYN-1
3X FLAG::RDE-3; MUT-15::3X HA; 3X V5::NYN-2

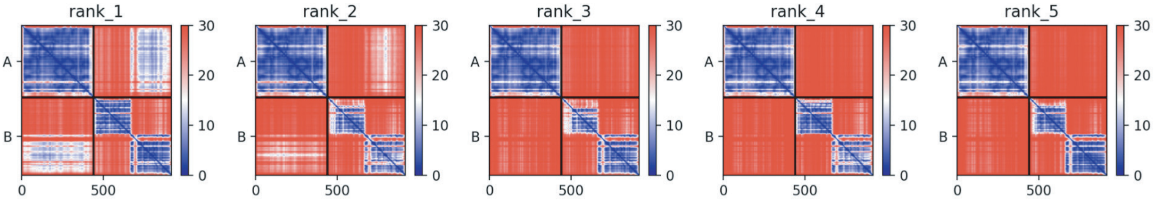

A: RDE-3 B: MUT-15(Δ2-44)

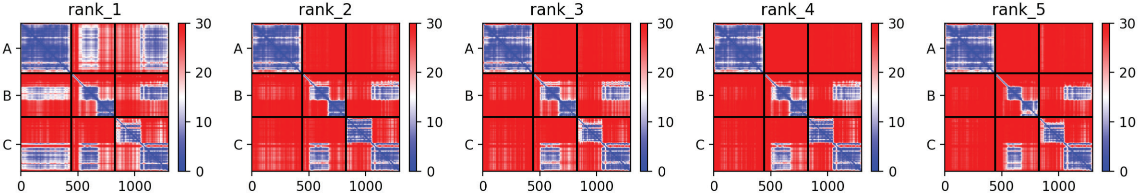

A: RDE-3 B: MUT-16(strd) C: MUT-15(Δ2-44)

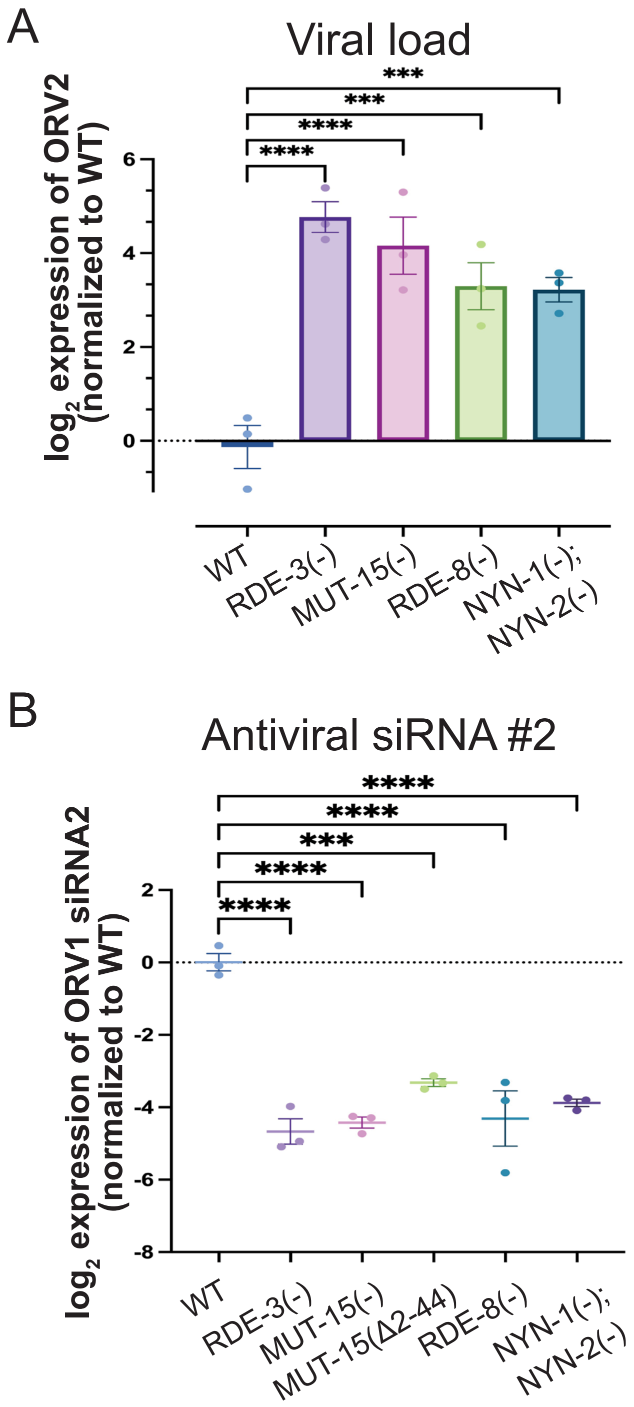
A
Viral load
***
***
****
****
6
4
2
0
log2 expression of ORV2
(normalized to WT)
WT
RDE-3(-)
MUT-15(-)
RDE-8(-)
NYN-1(-);
NYN-2(-)
B
Antiviral siRNA #2
****
****
***
****
****
2
0
-2
-4
-6
-8
log2 expression of ORV1 siRNA2
(normalized to WT)
WT
RDE-3(-)
MUT-15(-)
MUT-15(Δ2-44)
RDE-8(-)
NYN-1(-);
NYN-2(-)
