# Supplementary material for: Viral RNA pUGylation promotes antiviral immunity in C. elegans
Source: J Virol. 2025 Oct 30;99(11):e01169-25. doi: 10.1128/jvi.01169-25 (PMC12645942; doi:10.1128/jvi.01169-25)
Supplement: Supplemental legends — Legends for Figures S1 to S13. [file jvi.01169-25-s0005.pdf]

Figure S1. Auxin-dependent depletion of RDE-3. Animals of the indicated genotypes were exposed to *dpy-6* RNAi +/- auxin. Animals that failed to respond to *dpy-6* RNAi exhibit normal body sizes, while animals that respond to *dpy-6* RNAi exhibit a Dumpy phenotype.

Figure S2. Schematic for detecting pUG RNAs and small RNAs. (A) General schematic for detecting pUG RNAs for quantitative-PCR and gel-based qualitative PCR. Purified RNAs are reverse-transcribed with a poly(AC)-primer to generate cDNAs, which are then amplified using a nested PCR primer. The amplified products are treated with Exo I and diluted for a second round of PCR for gel analysis or quantification on a qPCR machine. **(B)** General schematic for Taqman assay to detect small RNAs. Purified RNAs are reverse-transcribed using a stem-loop primer that is specific to each small RNA. qPCR is performed with custom primers targeting each small RNA and a Taqman probe, which contains a fluorophore and quencher. During amplification, the polymerase will chew away the probe to release the fluorophore and promote fluorescence for quantitative measurements.

Figure S3. Spike-in RNA controls for pUG-Seq. *In vitro* transcribed gfp(UG)18 RNA was spiked-in to RNA preparations during library preparation. Nanopore sequencing revealed that WT and RDE-3(-) animals had similar levels of spiked-in pUG RNA over three replicates. Unpaired, two-tailed t-test. n=3. ns - not significant,* $p<0.05$, ** $p<0.01$, *** $p<0.001$, **** $p<0.0001$.

Figure S4. PAE and 3D representations of Alphafold2 predicted interactions between RDE-3, MUT-15, and MUT-16. (A) (Top) A predicted 3D structure of the top ranked Alphafold2 prediction for RDE-3 and MUT-15. (Bottom) Five independent PAE plots for the predicted RDE-3-MUT-15 interaction. Rank #1 is also shown in Figure 3. **(B)** Five independent PAE plots for the predicted full-length MUT-16 and MUT-15 interaction. **(C)** (Top) The top PAE plot for the predicted RDE-3-MUT-15-MUT-16(strd) predicted interaction is shown. (Middle) A predicted 3D structure (rotated 180°) of the top ranked prediction of the trimeric complex of RDE-3-MUT-15-MUT-16(strd). Yellow lines connect sections of PAE plots (yellow rectangles) to relevant sections of 3D structures (yellow circles). (Bottom) Five independent PAE plots for RDE-3-MUT-15-MUT-16(strd). PAE plots are color-coded to show predicted distances between residues: 0 Angstroms (blue) to 30 or greater Angstroms (red).

Figure S5. PAE and 3D representations of Alphafold2 predicted interactions between MUT-15, NYN-1/2, and RDE-8. (A) Five independent PAE plots for the predicted (top) MUT-15-NYN-1 and (bottom) MUT-15-NYN-2 interactions. **(B)** (Top) Predicted 3D structures and PAE plots for (left) RDE-8 (yellow) and (right) RDE-8 (yellow) and NYN-2 (orange). Red circles and red-colored residues indicate the RDE-8 catalytic site. Corresponding PAE plots are shown. The catalytic site of RDE-8 is labeled in red. PAE plots are measured in Angstroms: 0 (red) to 30 (blue). **(C)** Five independent PAE plots for the predicted (top) RDE-8-NYN-1 and (bottom) MUT-15-NYN-2 interactions. PAE plots are color-coded to show predicted distances between residues: 0 Angstroms (blue) to 30 or greater Angstroms (red).

Figure S6. Alphafold2 predicts a heteropentamer pUGasome complex. (Top) The top PAE plots for the predicted MUT-16(strd), RDE-3, MUT-15, RDE-8, and NYN-1 or NYN-2 interaction. (Middle) Predicted 3D structures of pentameric complexes containing NYN-1 (left) and NYN-2 (right). Two images are shown for each prediction, rotated 180°. Inset: RDE-3 catalytic site residues shown in red. (Bottom) Five independent PAE plots for the predicted pentameric complexes with NYN-1 or NYN-2. PAE plots are color-coded to show predicted distances between residues: 0 Angstroms (blue) to 30 or greater Angstroms (red).

Figure S7. The predicted pUGasome without MUT-16. As described in Figure S6, except MUT-16(strd) was omitted from the Alphafold2 prediction and only a NYN-1 containing 3D representation is shown. The NYN-2 predicted structure was similar.

Figure S8. Epitope tags do not disrupt RDE-3, RDE-8, and MUT-15 functions in RNAi. (A) This panel shows that loss-of-function mutations in the predicted components of the pUGasome are, as expected, defective for dpy-6 RNAi. Animals of the indicated genotypes were exposed to +/- *dpy-6* RNAi. Animals that failed to respond to *dpy-6* RNAi exhibit normal body sizes, while animals that respond to *dpy-6* RNAi exhibit a Dumpy phenotype. **(B)** This panel shows that introduction of epitope tags to the endogenous copies of *rde-8, rde-3, and mut-15* do not disrupt the function of the encoded proteins in *dpy-6* RNAi.

Figure S9. MUT-15(Δ2–44) is not strongly predicted to interact with RDE-3. Four of five PAE plots for RDE-3-MUT-15(Δ2–44) and RDE-3-MUT-15(Δ2–44)-MUT-16(strd) do not predict an

interaction. PAE plots are color-coded to show predicted distances between residues: 0 Angstroms (blue) to 30 or greater Angstroms (red).

Figure S10. The predicted pUGasome components are required for antiviral immunity. The data in this figure show that the predicted components of the pUGasome are required for antiviral immunity as measured by ORV2 levels and ORV1 siRNA #2 quantification. **(A-B)** Genotypes as in Figure 5. **(A)** qRT-PCR for ORV2 RNA levels. Data were normalized to mRNA controls *eft-2* and *cdc-42* and viral loads in WT were defined as zero. Unpaired, one-way ANOVA. n=3. **(B)** Taqman assays for antiviral ORV1 siRNA2. Data were normalized to the control *U18* snRNA and signals in WT were defined as zero. Paired, one-way ANOVA. n=3. ns - not significant,* p<0.05, ** p<0.01, *** p<0.001, **** p<0.0001.

Figure S11. MUT-16 and RdRP are needed for antiviral siRNA production and antiviral immunity. (A) qRT-PCR using RNA isolated from Orsay infected wild-type [WT], *mut-16(pk710)* [MUT-16(-)], and *rrf-1(pk1417); ego-1(gg685)* [RdRP(-)] animals to assess ORV2 viral load. Data are normalized to housekeeping genes *eft-2* and *cdc-42* and signals in WT were defined as one. Unpaired, one-way ANOVA. n=3 **(B)** Taqman assay using RNA isolated from Orsay infected animals of genotypes described in **(A)** detecting ORV1 siRNA #2. Data were normalized to the control *U18* snRNA and signals in WT were defined as zero. Paired, one-way ANOVA. n=3. ns - not significant,* p<0.05, ** p<0.01, *** p<0.001, **** p<0.0001.

Figure S12. Alphafold2 prediction of RdRP-MUT-16 interaction. Five independent PAE plots for Alphafold2 predicted interactions between **(A)** RRF-1-EKL-1, **(B)** MUT-16-EKL-1, **(C)** MUT-16-RRF-1 and **(D)** MUT-16(484-631)-RRF-1-EKL-1. **(E)** Predicted 3D structure of the top ranked Alphafold prediction for the MUT-16(484-631)-RRF-1-EKL-1 interaction, along with the corresponding PAE plot. PAE plots are color-coded to show predicted distances between residues: 0 Angstroms (blue) to 30 or greater Angstroms (red).

Figure S13. Surface electrostatic potential of the predicted pUGasome. (A-B) Electrostatic potential of the Alphafold2 predicted heteropentamer containing **(A)** RDE-3, RDE-8, NYN-2, MUT-15, and MUT-16(strd) and **(B)** RDE-3, RDE-8, NYN-2, and MUT-15. **(A-B)** Two predicted structures, rotated by 180°, are shown for each. **(C)** 3D representation of Alphafold2 prediction that included an

RNA in the prediction is shown. Electrostatic potential is indicated with positively-charged residues in red and negatively-charged residues in blue.
